# A Prospective Cohort Study on Pregnancy Outcomes of Persons Immunized with a Seasonal Quadrivalent Inactivated Influenza Vaccine during Pregnancy

**DOI:** 10.3390/vaccines10101577

**Published:** 2022-09-21

**Authors:** Christopher Robinson, Janine Oberye, Josephine van Boxmeer, Jessica D. Albano, Hugh Tilson, Anthony Scialli, John A. Vanchiere, Ellis Ides, Daphne Sawlwin, Matthew Hohenboken, Jonathan Edelman

**Affiliations:** 1 Charleston Maternal Fetal Medicine, Summerville, SC 29485, USA; 2 Seqirus Netherlands B.V., 1105 BJ Amsterdam, The Netherlands; 3 Syneos Health, Wilmington, NC 28403, USA; 4 Gillings School of Global Public Health, University of North Carolina, Chapel Hill, NC 27599, USA; 5 Scialli Consulting LLC, Washington, DC 20008, USA; 6 LSU Health Science Center, Shreveport, LA 71106, USA; 7 Seqirus Australia Pty Ltd., Parkville, VIC 3052, Australia; 8 Seqirus USA Inc., Cambridge, MA 02139, USA; 9 Seqirus USA Inc., Summit, NJ 07901, USA

**Keywords:** stillbirth, spontaneous abortion, preterm birth, low birthweight, major congenital malformations, influenza vaccination

## Abstract

This US-based, prospective observational cohort study evaluated the safety of a quadrivalent inactivated influenza vaccine (IIV4; Afluria Quadrivalent) in pregnant persons immunized over four influenza seasons between 2017 and 2021. Pregnancy outcomes included live birth, stillbirth, spontaneous abortion, and elective termination. Infant events of interest were major congenital malformations (MCMs), preterm birth (<37 weeks gestational age), and low birth weight (LBW). Data were descriptive; prevalence point estimates were reported with 95% confidence intervals (CI). A total of 483 pregnant persons were given IIV4 and evaluated; 477 (98.8%) reported a live birth, and there were 2 stillbirths, 4 spontaneous abortions, and no elective terminations or maternal deaths. The prevalence rates of infant events were as follows: preterm birth, 7.2% (upper 95% CI, 9.6%); LBW, 5.4% (upper 95% CI, 7.4%); and MCMs, 0.8% (upper 95% CI, 1.9%). Point estimates and upper 95% CIs of the observed prevalence rates were lower than or similar to background prevalence in the general US population. Our findings suggest no evidence of a safety concern with vaccinating this group at high risk of influenza complications and are consistent with published data from databases and surveillance systems that monitor the safety of influenza vaccines in pregnant persons.

## 1. Introduction

Influenza vaccination during pregnancy decreases the risk of influenza disease and its complications among pregnant persons and their infants [1]. Pregnant persons are at increased risk of morbidity and death from influenza—in a recent cross-sectional study of US influenza hospitalization data, one-third of reproductive-age persons hospitalized with laboratory-confirmed influenza were pregnant [2]. In addition, the risk of preterm birth and low birthweight (LBW) is higher among newborn infants when their mothers have influenza—especially severe illness—during pregnancy [3]. Moreover, maternal influenza vaccination has been shown to prevent influenza infection in infants 0–6 months of age, which is of relevance, as they are not eligible to receive influenza vaccines, yet those infected with influenza have the highest rates of hospitalization and death among all children [2,4,5]. For these reasons, the US Advisory Committee on Immunization Practices (ACIP) recommends a seasonal influenza vaccine for people who are or will be pregnant, among other groups at high risk from influenza, and the American College of Obstetricians and Gynecologists (ACOG) encourages persons to receive an influenza vaccine at any time during each pregnancy [6,7].

Recent estimates of influenza vaccination during pregnancy have shown that only 61% of pregnant people reported receiving an influenza vaccine, which is well short of the 80% Healthy People 2020 coverage target, and there is a coverage gap of ~15% between more and less advantaged socioeconomic groups [8,9,10]. A key reason for vaccine hesitancy among pregnant people is concern for the fetus [11]. Although the safety of influenza vaccines during pregnancy is generally well documented [11,12,13,14,15], brand-specific safety data are limited.

A quadrivalent inactivated influenza vaccine (IIV4; Afluria Quadrivalent, Seqirus, Parkville, Australia) was first licensed for use in adults in 2016 in Australia and the US and has since been approved in New Zealand, Canada, Argentina, Germany, Austria, and South Korea. In this prospective observational study, we evaluated the safety of IIV4 in terms of adverse pregnancy outcomes and major congenital malformations (MCMs), preterm birth, and LBW in infants born to people who received IIV4 during pregnancy.

## 2. Materials and Methods

This prospective observational cohort study, a postmarketing commitment to the Food and Drug Administration (FDA), was conducted over four consecutive influenza seasons from 2017–2021. The Syneos Health Registry Coordinating Center (Wilmington, NC) served as the single site for the study, but data were collected from obstetrics/gynecology clinics that used IIV4 as a component of routine care and were willing to enroll pregnant people vaccinated with IIV4 in their clinic. Study participants could also self-enroll (e.g., after reading about the study in medication package inserts or other media).

The schedule of visits, laboratory tests, all treatment regimens, and timing of immunizations in this strictly observational study were determined by the treating health care provider (HCP). This safety study did not include clinical assessments to evaluate influenza vaccine effectiveness. The study was designed and conducted in a manner consistent with the 2002 US FDA guidelines for pregnancy exposure registries, which was effective when the study started [16], as well as good pharmacoepidemiology practices, applicable local regulations, and the Declaration of Helsinki [17]. An institutional review board approved the protocol and consent form. All participants provided informed consent either in writing or verbally. Because this study involved routine care that posed no excess risk to participants, written consent was not required per US regulations. Protected health information and identifying information for all study participants were removed prior to data reporting.

### 2.1. Study Population

The study population included pregnant persons residing within the US who received IIV4 at any time as part of routine pregnancy care for whom sufficient data were available to confirm the subject was enrolled prospectively, the date and specified brand of IIV4 vaccination, pregnancy outcome, and whether an event of interest in the fetus or infant had occurred. Both passive and active recruitment strategies were implemented. Subjects were recruited by healthcare providers (HCP) from obstetrics/gynecology clinics that used IIV4 per routine care and were willing to enroll eligible and consenting pregnant persons vaccinated in their clinic. In addition, a targeted awareness campaign was undertaken that allowed subjects to self-enroll in the study. Subject selection and recruitment strategies are described in greater detail in the Supplementary Information. Comparison group data were not collected within the scope of this study, but background prevalence data from external surveillance sources and data from published literature were used to provide context.

### 2.2. Vaccination

IIV4 (Afluria Quadrivalent) is a purified, split, inactivated, egg-derived, nonadjuvanted, quadrivalent influenza virus vaccine formulated to contain hemagglutinin antigens from the A(H1N1), A(H3N2), B/Yamagata, and B/Victoria strains selected for each season by the World Health Organization (WHO). Vaccination was administered solely as part of routine clinical care at the discretion of the pregnant person and the treating HCP.

### 2.3. Pregnancy Outcomes and Events of Interest

Pregnancy outcomes of interest included live birth, stillbirth (fetal death occurring at ≥20 weeks of gestation or of a fetus weighing ≥500 g), spontaneous abortion (fetal death prior to 20 weeks of gestation), or elective termination. Ectopic pregnancy or molar pregnancy were reported as pregnancy types. Infant-related events of interest included preterm birth (<37 weeks gestational age), low birthweight (<2500 g), or MCM, which was defined and coded using criteria specified by the CDC Metropolitan Atlanta Congenital Defects Program (MACDP). Specifically, any major structural or chromosomal defect or combination of three or more minor defects in liveborn infants, stillbirths, or fetal losses of any gestational age (including outcomes prior to 20 weeks’ gestation or birthweight <500 g) was considered an MCM. Information on clusters of minor abnormalities (as defined by the CDC MACDP) and data from aborted fetuses of less than 20 weeks’ gestation, when available, was included to increase the sensitivity of monitoring. Infant size was also calculated using the 2006 WHO Child Growth Standards used to derive the z scores for weight-for-age and sex with outputs reported in percentile categories [18].

HCPs from participating clinics and/or pregnant persons or their legally authorized representatives served as data reporters for this study. The only data collected were those documented in the patient’s medical records as part of routine care; no assessments were required as part of the study. Data regarding events of interest were provided by the obstetric HCP and/or pediatrician attending the birth. Gestational age at birth and birthweight were used to assess preterm birth and LBW. A teratologist who was blinded to the timing of vaccination reviewed all reported congenital anomalies and classified them using the CDC MACDP system and provided an opinion regarding the gestational time window during which exposure to IIV4 could potentially have had an impact on the reported birth defect. A Scientific Advisory Committee (SAC), consisting of experts in the fields of obstetrics/maternal-fetal medicine, pediatrics, clinical research, infectious disease, epidemiology, and teratology, met periodically to review the data and make recommendations on the coding and classification of MCMs and other outcomes of interest.

### 2.4. Statistical Methods

This was a descriptive study, and a planned sample size of 550 (with an anticipated loss to follow-up of ~10%, for a final evaluable population of 500 pregnant persons) was not based on a statistical hypothesis to be tested but instead was determined based on the US background prevalence of MCMs (2.8%), preterm births (11.4%), and LBW (8.0%) according to the CDC MACDP and National Vital Statistics System (NVSS), as available at the time the study was designed [19,20]. Assuming 166 first-trimester exposure pregnancies [21], the study was expected to have at least 80% power to detect a 2.5-fold increase in the prevalence of MCMs and >99% power to detect a statistically significant 2.0-fold increase in preterm births or LBW.

Pregnancy outcomes and events of interest were summarized overall, by trimester of exposure, and by maternal age at conception categories; 2-sided 95% confidence intervals (CIs) were calculated for pregnancy outcomes using the exact binomial distribution (Clopper–Pearson method). The prevalence of MCMs was calculated by including all MCMs in liveborn infants as well as MCMs in stillborn infants with a gestational age of ≥20 weeks in the numerator and including all live births in the denominator. Overall and stratum-specific prevalence point estimates and 1-sided 95% CIs were calculated using the exact binomial distribution for prevalence of MCMs among all evaluable live births, for prevalence of LBW among evaluable pregnant persons with a singleton live birth without MCMs, and for prevalence of preterm birth among evaluable persons enrolled prior to 37 weeks’ gestation with a singleton live birth without MCMs. Birth outcomes were compared to the most recently reported corresponding prevalence estimates from the CDC MACDP and NVSS [19,22].

## 3. Results

### 3.1. Demographic and Clinical Characteristics of Enrolled Pregnant Persons

**Figure 1:**
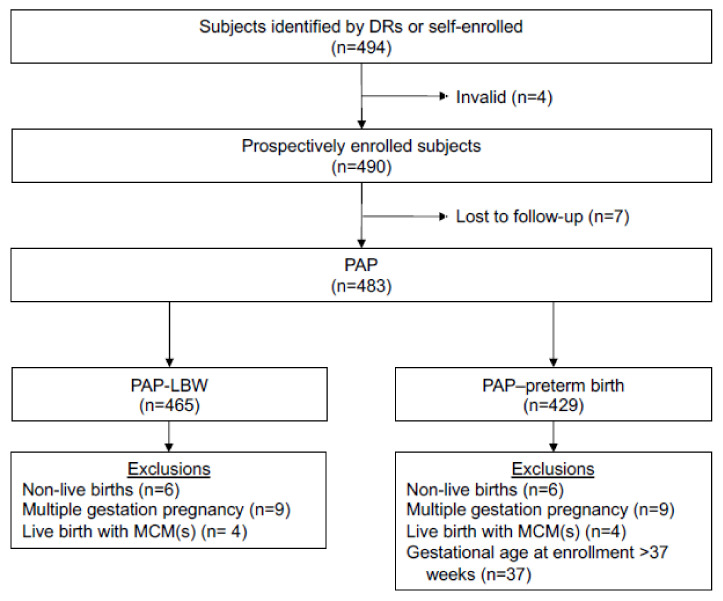
Flow diagram of the overall study population and exposure. HCP, healthcare provider at participant obstetrics/gynecology clinic; LBW, low birth weight; MCM, major congenital malformation; PAP, primary analysis population. For the 483 pregnant persons in the PAP, infant outcome data were reported for 485 infants; 9 pregnant persons carried twin pregnancies, of which one resulted in a spontaneous abortion; and there were 2 stillbirths and 3 spontaneous abortions among the singleton pregnancies. Subjects excluded from the PAP-LBW and/or PAP-preterm birth can be counted in more than 1 category.

**Figure 2:**
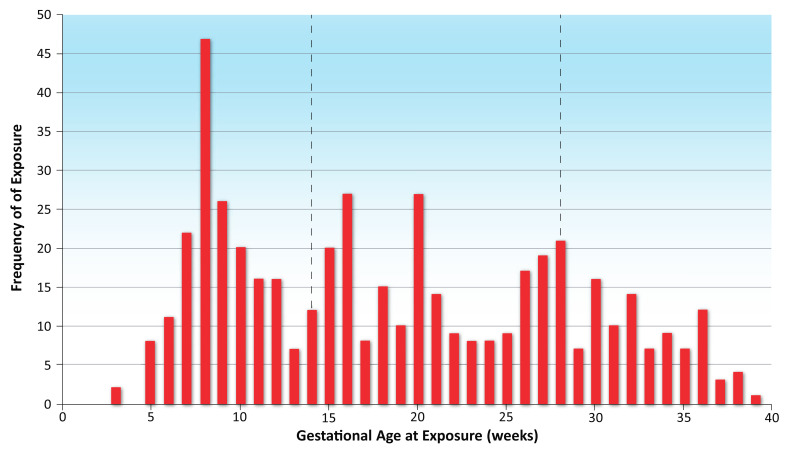
Exposure to quadrivalent inactivated influenza vaccine (IIV4) per gestational week for all enrolled subjects (*n* = 490). The dotted lines at week 14 and week 28 represent the beginning of the second and third trimesters, respectively.

Between 2017 and 2021, 494 participants were enrolled, of whom 4 were subsequently found to be ineligible. The 490 remaining pregnant persons were either self-enrolled (*n* = 6) or enrolled at one of five participating clinics in New York and North Carolina (*n* = 484) (Figure 1). Influenza vaccinations were received by 175 (35.7%) of the prospectively enrolled pregnant persons during the first trimester, 203 (41.4%) during the second trimester, and 111 (22.7%) during the third trimester (Figure 2). The primary analysis population comprised 483 pregnant persons; 7 (1.4%) enrolled persons were lost to follow-up (Table S1).

The mean maternal age at conception was 29.8 ± 5.4 years (Table 1). The study population was 67.7% White, 14.5% Black, and 12.8% Asian; 76.4% had at least one concurrent condition, and 97.7% used at least one concomitant medication (most commonly prenatal vitamins).

### 3.2. Pregnancy Outcomes

Overall, 477 (98.8%) pregnancies ended in a live birth, including 97.1, 99.5, and 100% of pregnancies with exposure to IIV4 during the first, second, and third trimesters, respectively (Table 2). Two stillbirths were reported: one after a first-trimester exposure and one after a second-trimester exposure. Among the 160 pregnant persons enrolled before 20 weeks of gestation, there were four spontaneous abortions (2.5%), one of which involved twins, and no elective terminations. All spontaneous abortions occurred in persons 25–34 years of age and who were exposed in the first trimester. No ectopic pregnancies or molar pregnancies were reported, nor were there any maternal deaths.

### 3.3. Infant Events of Interest

The infant primary analysis population for the calculation of the prevalence of MCMs included 485 liveborn infants from 483 pregnant persons. Nine carried twin pregnancies, of which one resulted in a spontaneous abortion, and there were two stillbirths and three spontaneous abortions among the singleton pregnancies. After excluding the 16 liveborn infants from the remaining twin pregnancies and four infants with MCMs, the LBW analysis population included 465 infants (Figure 1). The preterm birth analysis population included 429 infants after excluding infants born after IIV4 exposure at gestational age >37 weeks in addition to the aforementioned exclusions for the LBW population (Figure 1).

The infant outcomes appear in Table 3. Overall, 263 (54.2%) of the infants were male, and 221 (45.6%) were female. The mean (±SD) birthweight and gestational age at pregnancy outcome were 3341.3 ± 536.1 g and 38.7 ± 1.63 weeks, respectively. No infant deaths were reported. LBW was reported for 25 infants (5.4%, upper 95% CI, 7.4%). Using the WHO international standard, infant size was below the 15th percentile for 86 of 485 infants (17.7%). Preterm birth was reported for 31 infants (7.2%, upper 95% CI, 9.6%). HCPs reported five infants with an MCM, of which four cases (gastroschisis (two cases), lymphangioma, syndactyly) were adjudicated by the SAC as being MACDP-defined MCMs, for an overall prevalence of 0.8% (upper 95% CI, 1.9%). The remaining HCP-reported MCM (hypoxic ischemic encephalopathy) was adjudicated by the SAC as not being an MACDP-defined MCM. The two infants with gastroschisis were born to mothers with a first-trimester exposure; the infants with lymphangioma and syndactyly were born to mothers with a second-trimester exposure. SAC review determined that a temporal association with IIV4 exposure could not be ruled out for the two gastroschisis cases, too little information was available to assess temporality for the case of lymphangioma, and there was no temporal association for the case of syndactyly. Potential confounding factors noted by the teratologist for the two gastroschisis cases included young maternal age and urinary tract infection; no confounding factors were noted for the lymphangioma and syndactyly cases.

## 4. Discussion

Our findings are consistent with previous studies looking at all influenza vaccines combined (i.e., irrespective of type and brand), showing that maternal immunization does not increase the risk of adverse pregnancy outcomes [7,11,12,13,14,15,23]. In this study of 483 pregnant persons vaccinated with IIV4 between 2017 and 2021, the live birth rate was >98%, and adverse maternal and infant outcomes were comparable to—or even lower than—national averages. Two stillbirths were reported, and the estimated rate of 0.4% (95% CI, 0.1% to 1.5%) in this study is consistent with the 2017 US fetal mortality rate of 0.6% [24]. The rate of spontaneous abortion—2.5% (95% CI, 0.7% to 6.3%)—is lower than the 10% estimate from the ACOG for early pregnancy loss [25], even though only pregnant persons enrolled before 20 weeks’ gestation were included in the calculation of the prevalence of spontaneous abortion. Moreover, few pregnant persons were vaccinated in their first trimester (Figure 2), when the risk of spontaneous abortion was highest.

In 2020, the NVSS reported a preterm birth rate of 10.1% in the US [22]. The estimated prevalence in this study (7.2%; upper 95% CI, 9.6%) falls below this population statistic. The NVSS reported the prevalence of LBW as 8.2% for all births and 6.7% for singletons in 2020 [22]; in this study, the rate was 5.4% with an upper 95% CI of 7.4%, which is consistent with both rates reported for the general US population. Importantly, the rate of MCMs (0.8%, upper 95% CI, 1.9%) in this study is lower than the most recently reported overall rate of 2.8% [19]. These study data suggest that there is no evidence of a teratogenic effect of IIV4 during pregnancy.

Although the pregnancy registry was not designed or powered to investigate specific malformations, there were two cases of gastroschisis among the four reported MCMs that merit further discussion. Gastroschisis is the most common type of congenital abdominal wall defect in infants. Its prevalence has increased over the last three decades, with the highest prevalence observed among mothers <20 years of age (15.7 per 10,000 live births) [26,27,28,29]. Approximately 70% of infants with gastroschisis are born to persons <25 years of age, and persons younger than 20 years are more than 7 times more likely to have an offspring with gastroschisis than persons aged 25–29 years [29,30]. The mechanism underlying gastroschisis is not well understood, and experts have not reached consensus on, among other factors, the gestational age window during which gastroschisis is thought to occur, which varies from 3–5 weeks to 6–11 weeks postconception [29]. However, the risk factors for this congenital anomaly are well described and include young maternal age, smoking, illicit drug use, antipsychotic medications, alcohol consumption, low maternal body mass index, and genitourinary tract infections [29,31,32,33]. In the current study, the SAC concluded that a temporal association with IIV4 exposure in the two cases of gastroschisis could not be ruled out, but noted young maternal age as a contributing factor in one case and urinary tract infection as a contributing factor in the other. Based on the sample size of the current study, the possibility exists that the reporting of two cases of gastroschisis in this study population is a chance finding, since no other cases of gastroschisis have been reported spontaneously in the pharmacovigilance database for IIV4.

## 5. Limitations

Most registry participants were enrolled at one of five obstetrics/gynecology clinics in the eastern US, and a larger sample size would be required for full representation of the population of pregnant persons in the US. Despite these limitations, the study population included the most common racial and ethnic groups in the US, in similar proportions to the general population. In addition, the study population encompassed a broad range of maternal ages (18–43 years) and a relatively even distribution in the number (0 to ≥4) of previous pregnancies. Approximately 35% of registry participants were exposed to IIV4 in the first trimester, which is important for the assessment of maternal exposures and birth defects. High-risk pregnancies were not specifically excluded from the study, although there is a chance that very high-risk pregnancies may not have been under the prenatal care of the routine obstetrics/gynecology clinics from which the pregnant persons were enrolled. Particularly, in comparison to other pregnancy registries reported for branded influenza vaccines [34,35], the number of persons who were lost to follow-up was low (seven persons; 1.4%). Nevertheless, four of them left the care of the participating obstetrics/gynecology clinic, and it is possible that such patients left to seek more specialized care related to a high-risk pregnancy. As a result, it cannot be assumed that the pregnancy outcome data would show a similar pattern in the lost to follow-up population. The study also excluded pregnant persons who were known to have actual or perceived knowledge of an adverse pregnancy outcome at the time of enrollment to avoid selection bias toward more adverse pregnancy outcomes or events of interest, particularly MCMs, which can be detected early in pregnancy through prenatal testing. However, this exclusion could potentially have had the opposite effect and biased the results toward lowering the overall risk of MCMs in the study population. The sample size of the study was based on the overall prevalence of MCMs from historical data, and the study was not designed to examine the effect of potential confounders and effect modifiers, such as previous pregnancy outcomes, pregnancy complications, concurrent conditions, and concomitant exposures; thus, no conclusions can be drawn with respect to these potential confounders.

A general limitation of noninterventional studies in which HCPs collect data during routine care is the potential for missing data, including a limited level of detail, lower precision of measures reported, and potential ascertainment bias of adverse pregnancy outcomes (e.g., the reporting HCP may not know the condition of an aborted fetus and/or additional data such as the results of genetic testing or autopsy data). Finally, this study did not use an internal comparator group but compared the findings in the exposed study population to historical population statistics from the general US population. Potential differences in the characteristics of the underlying populations cannot be ruled out. Although the study used the same case definitions for MCMs as the MACDP, there were important differences in data collection. As an active surveillance program, the MACDP includes all defects reported in the defined region, whereas participation in this study was voluntary. Furthermore, the MACDP counts any MCMs that are detected in an infant up to the age of 6 years, whereas follow-up in this study ended upon completion of the pregnancy. As a consequence, major MCMs not immediately detectable at birth might have been missed.

## 6. Conclusions

The findings of this prospective safety study suggest that IIV4 exposure during pregnancy is not associated with any safety concerns based on comparison of the study results to background prevalence rates reported by the NVSS and MACDP. Our findings are consistent with published data from various databases and surveillance systems that monitor the safety of influenza vaccines in pregnant persons, further supporting use of influenza vaccines in this group at high risk from influenza.

## Figures and Tables

**Table 1:** Baseline demographic and clinical characteristics.

Characteristic	Primary Analysis Population (*n* = 483)
Mean age ± SD (range), years	
Maternal (i.e., pregnant person)	29.8 ± 5.4 (18–43)
Paternal	32.7 ± 6.2 (18–59)
Age group, *n* (%)	
<20 years	19 (3.9)
20–24 years	66 (13.7)
25–34 years	311 (64.4)
35–39 years	69 (14.3)
≥40 years	18 (3.7)
Race and ethnicity, *n* (%)	
White	327 (67.7)
Black or African American	70 (14.5)
Asian	62 (12.8)
American Indian or Alaskan Native	0
Native Hawaiian or other Pacific Islander	0
Other	21 (4.3)
Unknown	3 (0.6)
Hispanic or Latino	26 (5.4)
Mean body mass index ± SD, kg/m^2^	27.2 ± 6.8
Number of previous pregnancies, *n* (%)	
0	178 (36.9)
1	132 (27.3)
2	87 (18.0)
≥3	86 (17.8)
Family history of congenital malformations, *n* (%)	
Offspring	6 (1.2)
Maternal history	16 (3.3)
Paternal history	7 (1.4)
Any family history	29 (6.0)
Any concurrent condition, *n* (%)	369 (76.4)
Any concomitant medication, *n* (%)	472 (97.7)
Substance use, *n* (%)	
Tobacco	22 (4.6)
Alcohol	1 (0.2)
Illicit drug	5 (1.0)
Trimester of exposure, *n* (%)	
First	171 (35.4)
Second	201 (41.6)
Third	111 (23.0)
Gestational age	
At exposure, mean age ± SD, days	134.6 ± 65.3
<20 weeks at enrollment, *n* (%)	160 (33.1)
≥20 weeks at enrollment, *n* (%)	323 (66.9)

SD, standard deviation.

**Table 2:** Pregnancy outcomes by maternal age at conception and trimester of IIV4 exposure.

Outcome, *n* (%) (95% CI)	Overall (*n* = 483)	Maternal Age at Conception (Years)					Trimester of IIV4 Exposure		
		<20 (*n* = 19)	20–24 (*n* = 66)	25–34 (*n* = 311)	35–39 (*n* = 69)	≥40 (*n* = 18)	First (*n* = 171)	Second (*n* = 201)	Third (*n* = 111)
Live birth	477 (98.8) (97.3–99.5)	19 (100) (82.4–100)	66 (100) (94.6–100)	306 (98.4) (96.3–99.5)	69 (100) (94.8–100)	17 (94.4) (72.7–99.9)	166 (97.1) (93.3–99.0)	200 (99.5) (97.3–100)	111 (100) (96.7–100)
Stillbirth	2 (0.4) (0.1–1.5)	0 (0.0–17.6)	0 (0.0–5.4)	1 (0.3) (0.0–1.8)	0 (0.0–5.2)	1 (5.6) (0.1–27.3)	1 (0.6) (0.0–3.2)	1 (0.5) (0.0–2.7)	0 (0.0–3.3)

CI, confidence interval; IIV4, quadrivalent inactivated influenza vaccine (Afluria Quadrivalent).

**Table 3:** Infant outcomes by trimester of maternal exposure to IIV4.

	Overall (*n* = 485)	First (*n* = 171)	Second (*n* = 203)	Third (*n* = 111)
Gender, *n* (%)				
Male	263 (54.2)	87 (50.9)	122 (60.1)	54 (48.6)
Female	221 (45.6)	84 (49.1)	80 (39.4)	57 (51.4)
Missing	1 (0.2)	0	1 (0.5)	0
Birthweight, g	*n* = 480	*n* = 168	*n* = 202	*n* = 110
Mean ± SD	3341.3 ± 536.1	3379.5 ± 547.8	3315.7 ± 534.8	3330.2 ± 521.6
Mean gestational age at outcome ± SD, weeks	38.7 ± 1.6	38.6 ± 1.6	38.6 ± 1.7	38.9 ± 1.6
Infant size, *n* (%)				
≤15th percentile	86 (17.7)	28 (16.4)	42 (20.7)	16 (14.4)
>15–50th percentile	108 (22.3)	38 (22.2)	37 (18.2)	33 (29.7)
>50–85th percentile	201 (41.4)	67 (39.2)	90 (44.3)	44 (39.6)
>85th percentile	84 (17.3)	35 (20.5)	32 (15.8)	17 (15.3)
Missing	6 (1.2)	3 (1.8)	2 (1.0)	1 (0.9)
Prevalence of preterm birth, denominator *	***n* = 429**	***n* = 152**	***n* = 192**	***n* = 85**
*n* (%) (95% upper CI)	31 (7.2) (9.6)	10 (6.6) (10.9)	17 (8.9) (13.0)	4 (4.7) (10.4)
Prevalence of low birthweight, denominator **	***n* = 465**	***n* = 159**	***n* = 195**	***n* = 111**
*n* (%) (95% upper CI)	25 (5.4) (7.4)	7 (4.4) (8.1)	13 (6.7) (10.4)	5 (4.5) (9.2)
**Prevalence of MCMs, denominator**	***n* = 485**	***n* = 171**	***n* = 203**	***n* = 111**
*n* (%) (95% upper CI)	4 (0.8) (1.9)	2 (1.2) (3.6)	2 (1.0) (3.1)	0 (2.7)

CI, confidence interval, IIV4, quadrivalent inactivated influenza vaccine (Afluria Quadrivalent); MCM, major congenital malformation; SD, standard deviation. * Excludes nonlive births, multiple gestation pregnancies, and live births with MCM. ** Excludes nonlive births, multiple gestation pregnancies, live births with MCM, and gestational age at enrollment > 37 weeks.

## Data Availability

Not applicable.

## Supplementary Materials

The following supporting information can be downloaded at: https://www.mdpi.com/article/10.3390/vaccines10101577/s1, Table S1: Baseline demographic and clinical characteristics of enrolled patients lost to follow-up.
