# Supplementary material for: A Prospective Cohort Study on Pregnancy Outcomes of Persons Immunized with a Seasonal Quadrivalent Inactivated Influenza Vaccine during Pregnancy
Source: Vaccines (Basel). 2022 Sep 21;10(10):1577. doi: 10.3390/vaccines10101577 (PMC9611467; doi:10.3390/vaccines10101577)
Supplement: Supplementary file 1 [file vaccines-10-01577-s001.zip › vaccines-1877506 supplementary.pdf]

Study Population Selection

The minimum eligibility criteria required for enrollment were as follows:

- Sufficient information to confirm that the exposure of interest occurred during pregnancy and on which date.
- Sufficient information to determine whether the exposure concerned a prospectively enrolled subject since retrospective cases were ineligible for enrollment. Prospective enrollment is defined as enrollment of a pregnant exposed person prior to knowledge or perceived knowledge of the pregnancy outcome (e.g., structural defect or genetic abnormality noted on a prenatal test). Those with no abnormalities identified on a prenatal test prior to enrollment were considered prospectively enrolled.
- Reporter (e.g., HCP) contact information was required to allow for follow-up.

Recruitment Strategies

Study enrollment was open to all eligible pregnant persons; however, a number of activities were implemented to increase the enrollment rate. Both passive and active recruitment strategies were implemented. One recruitment strategy targeted HCPs who were known to immunize pregnant persons with the Afluria QIV vaccine. Additionally, a targeted awareness campaign was undertaken. This campaign included the distribution of a comprehensive informational kit designed to support HCPs in soliciting interest in study participation among pregnant persons. All messaging was in line with approved US product labeling of Afluria QIV. The kit included

- A brochure that briefly described the study purpose and procedures
- Enrollment form and sample informed consent form
- Important Safety Information
- Participant consent to contact card, which enabled the CC to contact the potentially participating persons and provide additional information about the study

A variety of other approaches to provide information to pregnant persons and their HCPs for possible enrollment in the pregnancy registry were also used:

- Internet:
    - FDA listing of pregnancy registries on www.fda.gov
    - www.clinicaltrials.gov
    - Society for Maternal Fetal Medicine listing of registries
    - Immunizationforwomen.org "Monitoring the Safety of Influenza Vaccination during Pregnancy"
    - REPROTOX®
    - Seqirus US website
- Print:
    - Afluria QIV package insert
    - Afluria QIV medication guide
- Education:
    - Active outreach to obstetric HCPs who routinely used Afluria QIV in their practice

Table S1. Baseline demographic and clinical characteristics of enrolled patients lost to follow-up.

Characteristic	Lost to follow-up (*n* = 7) *
**Mean age ± SD, years**	
Maternal (i.e., pregnant person)	33.4 ± 5.3
Paternal	32.5 ± 0.7
**Age group, *n* (%)**	
<20 years	0
20-24 years	0
25-34 years	5 (71.4)
35-39 years	1 (14.3)
≥40 years	1 (14.3)
**Race and ethnicity, *n* (%)**	
White	2 (28.6)
Black or African American	0
Asian	4 (57.1)
American Indian or Alaskan Native	0
Native Hawaiian or Other Pacific Islander	0
Other	0
Unknown	1 (14.3)
Hispanic or Latino	2 (28.6)
Mean body mass index ± SD, kg/m^2	21.8 ± 3.4
**Number of previous pregnancies, *n* (%)**	
0	1 (14.3)
1	2 (28.6)
2	0
≥3	3 (42.9)
**Family history of congenital malformations, *n* (%)**	
Offspring	0
Maternal history	0
Paternal history	0
Any family history	0
Any concurrent condition, *n* (%)	3 (42.9)
Any concomitant medication, *n* (%)	5 (71.4)
**Substance use, *n* (%)**	
Tobacco	0
Alcohol	0
Illicit drug	0
**Trimester of exposure, *n* (%)**	
First	4 (2.3)
Second	2 (1.0)
Third	0
**Gestational age**	
At exposure, mean age ± SD, days	109.0 ± 65.2
<20 weeks at enrollment, *n* (%)	3 (42.9)
≥20 weeks at enrollment, *n* (%)	3 (42.9)

* Information on the number of previous pregnancies, on all categories of family history of congenital malformations and on substance use was missing for 1 subject.
